# Addressing the needs of terminally-ill patients in Bosnia-Herzegovina: patients’ perceptions and expectations

**DOI:** 10.1186/s12904-018-0377-2

**Published:** 2018-11-19

**Authors:** S. Aebischer Perone, R. Nikolic, R. Lazic, E. Dropic, T. Vogel, B. Lab, S. Lachat, P. Hudelson, C. Matis, S. Pautex, F. Chappuis

**Affiliations:** 1 Division of Tropical and Humanitarian Medicine, Geneva University Hospitals, Rue Gabrielle-Perret-Gentil 6, 1205 Geneva, Switzerland; 2 Primary Health Care Center, Dom zdravja Doboj, Nemanjina 18, 74000 Doboj, Bosnia and Herzegovina; 3 Fondacija fami, Kralja Aleksandra 52, 74000 Doboj, Bosnia and Herzegovina; 4 Division of Tropical and Humanitarian Medicine, Geneva University Hospitals, Avenue de Beau-Séjour 22, Geneva, Switzerland; 5 Transcultural consultation and interpretation, Geneva University Hospitals, Rue Gabrielle-Perret-Gentil 4, 1211 Geneva 14, Switzerland; 6 Geriatrics and community palliative care unit, Geneva University Hospitals, Avenue Cardinal-Mermillod 36, 1227 Carouge, Switzerland

**Keywords:** Terminally ill patients, End of life care, Palliative care, Home care, Patients’ perceptions, Transition countries, Low and middle income countries, Bosnia-Herzegovina

## Abstract

**Background:**

Many terminally ill patients in Bosnia-Herzegovina (BiH) fail to receive needed medical attention and social support. In 2016 a primary healthcare centreer (PHCC) in Doboj (BiH) requested the methodological and technical support of a local partner (Fondacija fami) and the Geneva University Hospitals to address the needs of terminally ill patients living at home. In order to design acceptable, affordable and sustainable solutions, we involved patients and their families in exploring needs, barriers and available resources.

**Methods:**

We conducted interviews with 62 purposely selected patients using a semi-structured interview guide designed to elicit patients’ experiences, needs and expectations. Both qualitative and quantitative analyses were conducted, using an inductive thematic approach.

**Results:**

While patients were aware that their illnesses were incurable, they were poorly informed about medical and social support resources available to them. Family members appeared to be patients’ main source of support, and often suffered from exhaustion and financial strain. Patients expressed feelings of helplessness and lack of control over their health. They wanted more support from health professionals for pain and other symptom management, as well as for anxiety and depression. Patients who were bedridden or with reduced mobility expressed strong feelings of loneliness, social exclusion, and stigma from community members and – occasionally - from health workers.

**Conclusions:**

Our findings suggest a wide gap between patients’ end-of-life care needs and existing services. In order to address the medical, psychological and social needs of terminally ill patients, a multi-pronged approach is called for, including not only better symptom management through training of health professionals and improved access to medication and equipment, but also a coordinated inter-professional, inter-institutional and multi-stakeholder effort aimed at offering comprehensive medical, psycho-social, educational and spiritual support.

**Electronic supplementary material:**

The online version of this article (10.1186/s12904-018-0377-2) contains supplementary material, which is available to authorized users.

## Background

At any time, over 40 million individuals live with incurable diseases in need of palliative care [1]*.* More than three quarters of them (78%) live in low and middle-income countries with poor access to palliative care. As put forward by Mitchell [2], the organisation and delivery of palliative and end-of-life care (EoLC) must be improved globally and the World Health Organisation states in one of its resolutions, that EoLC should be integrated into mainstream health care [1]:

“To develop and strengthen, where appropriate, evidence-based guidelines on the integration of palliative care into national health systems, across disease groups and levels of care…”

EoLC includes medical treatment and support offered to patients with incurable, life-threatening, and/or chronic progressive diseases when curative care is no longer a primary objective. The aim is to provide patients with the best possible quality of life before death, while also providing appropriate support to their relatives. End of life care aims at preventing suffering and complications and includes medical treatment and care as well as psychological, social and spiritual support [3, 4].

Our study took place in Bosnia and Herzegovina (BiH), a former Yugoslav republic situated at the heart of the Balkans. In the post conflict reconstruction and through the health system reform, strengthening primary health care, based on the family medicine (FM) model, has been set as a priority. Currently more than 80% of municipalities of BiH are served by FM teams. However, palliative care services are limited to services provided by community nurses and two hospices in Tuzla and Ljubuski (*Personal communication, Samir Husic, chief of Palliative Care Center of Tuzla*) and the palliative home care department of the PHCC “Centar Sarajevo” covering the population of Sarajevo (*Personal communication Fuad Husic, director of PHCC of Sarajevo Canton*). In hospitals no beds are allocated for palliative care patients [5]. There are no other outpatients’ services organised in BIH for palliative care patients. Therefore, it appears that the range of services needs to be further extended to improve access to healthcare for elderly, patients with incurable conditions and terminally-ill patients who stay at home with little support or wait to be admitted in a hospital. Despite national laws stating that EoLC should be provided by primary care services [6–8] and hospital services [9], policies and resources are insufficient to guide the development of EoLC in BiH. EoLC are currently provided on an ad-hoc basis outside any existing system or community network.

The municipality of Doboj, northern BiH, covers a population of about 80′000 persons. Doboj has an above average need for EoLC, because its population has a relatively high proportion of people over 65 years of age (28%) [10], as compared to the EU (19.4%) [11]. High mortality from cardiovascular diseases (47%), cancer (22%) and metabolic and respiratory conditions (10%) [12] was recorded in all age groups in 2015.

Early 2016, the management of the municipal PHCC approached a local non-governmental organisation, the Fondacija fami, and the Geneva University Hospitals (HUG) with the intent to improve the type and level of services made available to terminally ill patients living at home. Indeed, research [13] demonstrated in rural communities a reduction in avoidable hospital admissions for patients at their end of life when cared by general practitioners.

Since little was known about the needs and perceptions of these patients in BiH, we undertook a qualitative study to investigate patients’ perceptions, expectations and proposals for improvement. This paper presents the results from interviews with patients as a first step of an ongoing program to improve care and support offered to terminally-ill patients in Doboj municipality.

## Methods

### Study design

We conducted semi-structured interviews with a purposely selected sample of 62 terminally ill patients, in order to explore their perceptions, experiences and expectations regarding their health care and social support.

### Setting and participants

The study was conducted in Doboj municipality between November and December 2016. To identify study participants, we used a 2-tier, purposive sampling strategy. We first asked 31 family medicine teams (FM teams) from Doboj PHCC (totalling 35 doctors and 68 nurses) to identify each up to 30 patients in need for EoLC. Inclusion criteria were: registered patients with incurable disease, over 18 years of age, and able to understand and answer the interview questions. Exclusion criteria were: patients unable to provide informed consent to participate in the study, or with any disability that impeded communication. A total of 900 patients were identified.

From each of the FM team’s list of patients, we chose and invited 2 patients to participate in the study, for a total of 62 patients. Individuals were chosen to reflect the geographical and demographic characteristics of terminally-ill patients in Doboj municipality. All 62 selected patients (31 male, 31 female) accepted to be interviewed. Forty-six were interviewed alone, while 16 were interviewed with the assistance of a family member. All but one were interviewed in their home. Weekly supportive debriefing was offered to the interviewers.

Table 1 summarizes participants’ characteristics.

**Table 1:** Characteristics of participants interviewed in Doboj municipality, Bosnia and Herzegovina (*n* = 62)

Gender	
Male	31
Female	31
Age (years)	
≤ 29	1
30–39	2
40–49	6
50–59	11
60–69	10
70–79	17
80–89	15
Place of interview	
Home	61
Field clinic	1
Education	
No formal education	5
Primary school	26
Secondary school	29
Higher education	2
Distance to health facility	
< 5 min	15
6–15 min	36
> 16 min	11

### Data collection

Based on preliminary exchanges and a review of relevant literature [14–17], we developed an interview guide consisting of 15 open-ended questions to explore patients’ experiences and expectations, investigating the following dimensions as shown in Table 2.

**Table 2:** Dimensions explored in the interview guide

1. Information about the health condition, right / desire to know 2. Dignity, respect 3. Autonomy (physical and functional) 4. Depression, anxiety 5. Tiredness/drowsiness 6. Respiratory distress 7. Pain 8. Nutrition, appetite 9. Nausea/vomiting 10. Other digestive problems: constipation/diarrhoea 11. Distress, fear 12. Spiritual needs and believes 13. Financial issues 14. Administrative issues 15. Family and concern about their future	

In addition to the open-ended questions, we also asked patients to rate their current perceptions about each of the 15 dimensions using a 4-points severity scale (See interview guide in Additional file 1).

The interviews were jointly conducted by two investigators: the project coordinator from the Fondacija fami and an appointed PHCC health worker, who noted down answers on hard copy interview guides in the local language. All interviews lasted between 45 and 90 min.

### Data analysis

Interview notes were anonymised, translated into English and entered in Epi-Info 7 to facilitate analysis.

Respondents’ answers to the open-ended questions were first grouped by interview question, and then themes within each question were identified by the interviewer from the Fondacija fami and a social scientist from the HUG. Descriptive codes were created to reflect emerging themes. Consensus about findings was reached over weekly conference calls and discussions among research team members from BiH and Switzerland.

### Ethics approval

The study was approved by the Geneva Canton Research Ethics Commission (CCER) (N°2016-01422) and the ethical board of the PHCC in Doboj. We required and obtained written consent from all patients prior to the interviews.

## Results

### Quantitative analyses

**Fig. 1:**
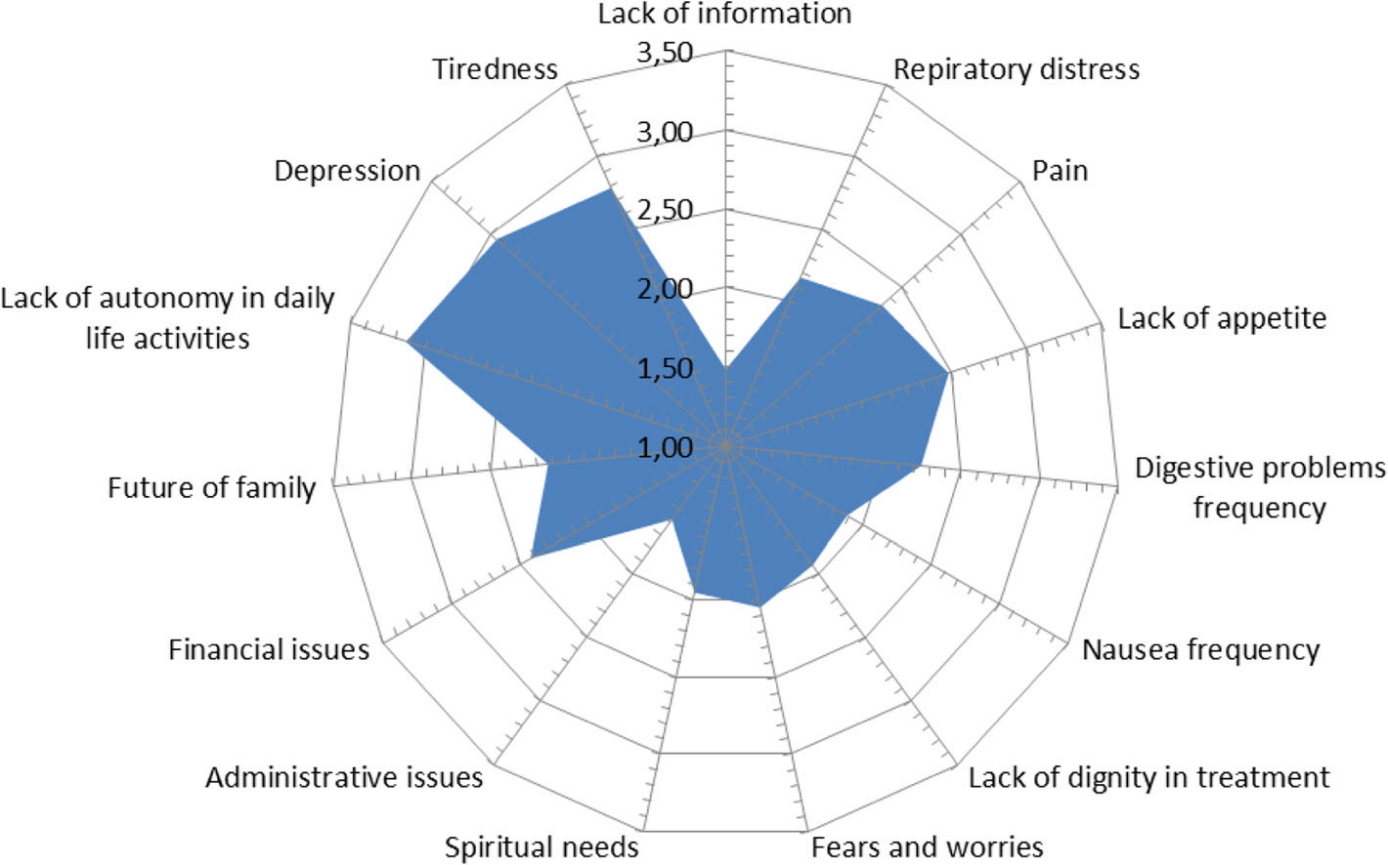
Fifteen themes and their relative weight according to patients

**Fig. 2:**
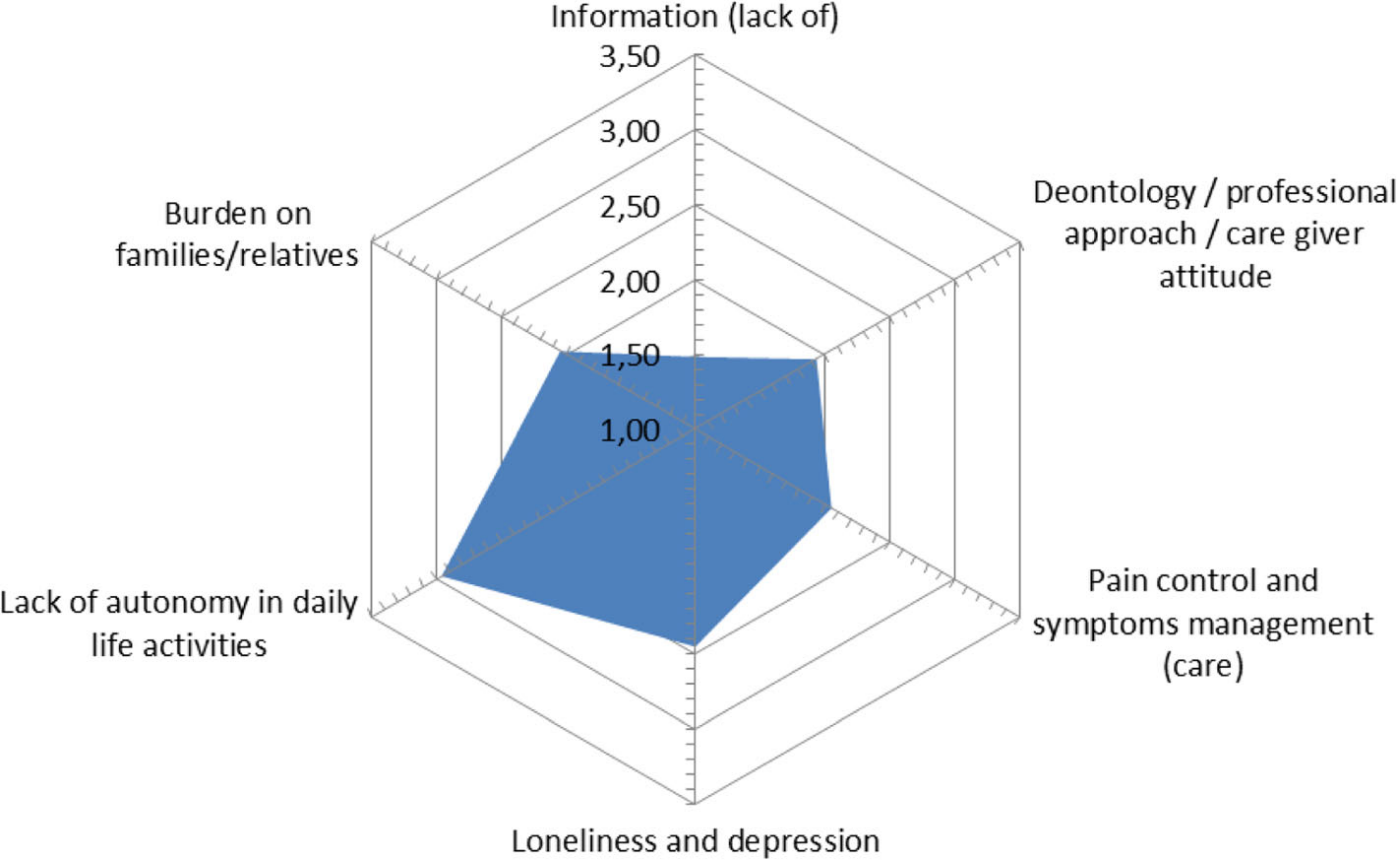
Emerging themes (grouping)

The quantitative analysis of the ratings given by the interviewed patients show – in decreasing order - the relative importance of: (i) lack of autonomy in daily activities, (ii) depression and loneliness, (iii) pain control and symptom management, (iv) burden on families, (v) deontology and professionalism and finally the (vi) lack of information. This analysis of the ratings confirmed the relative importance of the six identified themes as shown in the figures below (Figs. 1 and 2).

### Qualitative analysis

We took the emerging themes forward to proceed with a qualitative analysis that would provide further ground for the needs assessment and (future) interventions.

#### Theme 1 – Lack of autonomy

The majority of study participants (*n* = 50; 81%) depended entirely on their families for care and support and most persons mentioned being exhausted (*n* = 46).

- *«I depend on my family, especially my wife. I can just turn myself in the bed. My wife feeds me, I cannot get up, I lose my mind, I often forget things and have difficulties to speak. » (P6Q3a, M, 60–69, urban setting, lives with his relative)*
- *«I cannot move, pain exhausts me. I have no strength. I did not get help. They do not know how to help me. » (P11Q5a, M, 70–79, urban setting, lives with his relative)*

#### Theme 2 – Loneliness, depression and social exclusion

A majority of patients (*n* = 39; 63%) declared to be depressed (24 most of the time and 15 very often), particularly because of isolation and loneliness. The more restrained in their mobility and dependent they were, the more depressed they felt. Facing the deterioration of their condition and their impending death as well as painful symptoms were also sources of depression.

- *« We are both not well. I am exhausted and we both do not enjoy our lives any more. I wish we both die together and I wish it happens soon. » (P25Q4a, F, 50–59, urban setting, lives with her relative)*
- *« I cry, I lay down all the time in bed. This is no life any more. I do not enjoy my life at all. My husband as well. I wait for death and that is it. » (P28Q4a, F, 80–89, rural setting, lives with her relative)*

Some patients spontaneously stressed the need for psychological support and treatment against anxiety. Others explained that their ability to enjoy life could improve with increased social interaction, home visits by volunteers, peer support or even through technology-based interactions. Simply being able to go out from their bed or their home was also mentioned as a source of relief.

- *« We are isolated, people avoid us. Can people with this problem meet, or have volunteers to visit us and make us feel like human? » (P2Q4d, F, 80–89, urban setting, lives alone)*
- *« People know I am seriously ill and they either avoid me or feel sorry about me. I am not happy about it. It makes me forced to isolate myself from people. I am better off alone. » (P58Q2a, M, 60–69, rural setting, lives with his relative)*

Many patients (*n* = 27; 44%) found relief in faith, going to church, getting the communion and having the religious leaders visiting them.

- *« I believe in God and he helps me when I feel the worst. I have regular visits from the priest. » (P24Q12a, M, 30–39, urban setting, lives with his relative)*
- *« I did not solve those issues yet. Maybe I am angry at God because of all that has happened to me. I did not look for help. It is something I have to deal with myself. » (P18Q12a, M, 80–89, urban setting, lives with his relative)*

#### Theme 3 – Dissatisfaction with pain control and symptoms management

The majority of patients (*n* = 51; 82%) said they suffered from pain, and rated it from moderate (*n* = 21; 34%) to severe (*n* = 29; 47%) with one patient suffering from “the worst pain”. Almost all patients were treated with pain killers - including opioids - but reported that “it doesn’t help”. Many patients wished for home visits, some wanted stronger pain killers, others wanted physiotherapy or simply advice from specialists. Many were self-medicating with additional pain.

- *« My pain is strong and I would like sometimes to jump out of the window. Patients with such pain should have proper therapy. » (P1Q7c, F, 70–79, urban setting, lives with her relative)*
- *« I have strong pain. I buy pain killers. My doctor prescribes medication, but they help only for a short while. I become immune to this medication. I buy some pain killers that help my friends too. » (P16Q7a, F, 70–79, urban, lives with her relative)*

Most of the patients (*n* = 53; 85%) reported breathing difficulties, loss of appetite and weight (*n* = 53; 85%), digestive problems (*n* = 45; 73%), severe nausea (*n* = 15; 24%) often related to medication side effects (e.g. chemotherapy) or pain. In some cases (*n* = 3; 5%) the consequences of skin irritation from diarrhoea were worse than the diarrhoea itself. Some patients confirmed they were receiving medical treatment to address these problems but most were coping through self-adaptation (body position, diet, etc.) or by simply accepting that nothing could be done to improve their situation. Finally, more than two thirds of patients reported being exhausted (n = 45; 73%) due to their underlying condition or treatment.

- *« I did not ask for help. My disease influences my breathing. These breathing problems cause many difficulties. I know this is the consequence of my diagnosis. » (P13Q6a, F, 40–49, urban, lives with her relative)*
- *« I need support for my wounds, not for the diarrhoea. I need advice about what to do. I suffer because of diarrhoea’s consequences. » (P27Q10b, M, 50–59, urban setting, lives with his relative)*

#### Theme 4 -Burden on families

The majority of patients (*n* = 41; 66%) also worried about their family members and their future, and were afraid of becoming a burden on them. Most respondents (*n* = 43; 69%) expressed concern about the physical, social and financial strain on their family members as a result of their illness.

- *« My family worries more about me. I also worry about them. I am concerned on how they cope now and what will happen with them after I die. » (P27Q15a, M, 50–59, urban setting, lives with his relative)*
- *«I worry about my mum, she is exhausted. It is difficult to care for me. I worry about the financial situation of my family. I did not get sufficient support, just basic. » (P9Q15a, M, 40–49, rural setting, lives with his relative)*

Specifically, patients complained about the lack of access to paid caregivers, home based care, physiotherapy, wheelchairs or diapers.

- *« He could not even get a free wheelchair. He is neglected by the society. » (P14Q2a, M, 20–29, urban setting, lives with his relative)*
- *« Our families need better support, especially those who are all the time with us. My husband is exhausted. My daughter studies in Banja Luka and she comes from time to time. My husband takes care of everything. He works and earns for us. » (P13Q3b, F, 40–49, urban setting, lives with her relative)*

Only a few patients reported paying for full-time caregivers or nurses (day or night care) and some mentioned that in some circumstances the social services of Doboj paid for the caregivers. More than half of the patients (*n* = 32; 52%) mentioned financial difficulties when they needed to buy medicines, diapers or pay for caregivers. Only 14 (23%) did not have any financial issues. Most of the patients facing financial difficulties received support from their families and only few from social services and disability compensation.

#### Theme 5 – Deontology, professional approach and dignity of patients

One third of patients reported being mistreated, (*n* = 20; 32%), with five patients stating they had never been treated with dignity. Patients told of being refused care by emergency medical services “as they are dying anyway”, and of being left on their own, abandoned by friends and neighbours. More home visits were particularly desired.

- *« I do not want to go to the hospital. I feel bad there. They treat me bad, they just give me medication and nobody cares about me there. » (P18Q1c, M, 80–89, urban setting, lives with his relative)*
- *« Help patients have dignity in the last days of their life. We need better home care. » (P3Q2c, M, 80–89, urban setting, lives with his relative)*

However respondents benefitting from family support, friends, and sometimes medical staff reported being better off (*n* = 27; 44%). This helped them to gain a sense of dignity in the face of terminal illness and loss of autonomy.

- *« I am satisfied. The nurse comes once a month, she brings the doctor sometimes. The nurse is our blessing, we would be lost without her. » (P55Q1d, M, 50–59, rural setting, lives with his relative)*
- *« Thank God I had support from my daughter. I think that support only comes from the family. I feel bad about the patients who do not have family or lack their support. Every patient should have human support and understanding. » (P35Q2b, F, 70–79, urban setting, lives alone)*

#### Theme 6 – Lack of information

Almost all patients (*n* = 57; 92%) stated they were quite a bit (*n* = 19; 30%) or completely (38; 61%) informed about the course of their condition. However, a majority complained that nobody explained to them how to cope with their condition, feeling lost and left alone with their condition. Like their caregivers, they were asking for more support by health services and other public services, which – according to them – often do not pay enough attention. A few patients were not informed about their diagnosis, although they suspected the severity of their disease. It is important to stress that in most of these cases, the family members knew the diagnosis but did not inform the patient. Patients who were aware of their condition seemed better off:

- *« I have some problems with my brain. I do not know what the problem is. Something appeared on my CT. » (P26Q1a, M,60–69, urban setting, lives with his relative)*
- *« We are all lost. He is not talking or moving. He is completely paralyzed. Nobody told us what to do. He was sent home to die. We ask ourselves what to do now. » (P10Q1e, M, 50–59, rural setting, lives with his relative)*

Many (*n* = 19; 31%) patients reported a lack of information about available services. They reported challenges to access social and financial support (e.g. for diapers, catheters, care givers or transport to the medical referral centre). They also claimed a need for more information about health insurance, succession procedures, patients’ rights and general administrative procedures.

- *« It is difficult to have this disease. It is even more difficult to go through all procedures. This kind of patients are ill and still they have to organize everything themselves. » (P35Q1e, F, 70–79, urban setting, lives alone)*
- *« There should be a counselling service for cancer patients. Many people have cancer, it is like an epidemic. » (P36Q1f, M, 40–49, urban, lives with his relative)*

## Discussion

Our results suggest that in the absence of adequate services and support for terminally ill patients, symptom management may be unsatisfactory, patients may feel abandoned, and the burden of care may fall on family members who are challenged in taking on such a role. This situation can accentuate patients’ anxiety, and lead to financial and social problems for family members.

The exploration of patients’ needs and perceptions at the end of their life in Doboj reveals four key areas of concern.

Firstly, and as described by Sand [18], restricted social interaction caused by either loss of autonomy or by being left alone without adequate support led to isolation and loneliness for most patients. Patients with no relatives to take care of them appeared worse off. Feelings of neglect and stigma from community - but also from some health services or the society as a whole - were common, reported both by patients and caregivers. Home visits and home based care were asked for frequently.

Secondly, management of symptoms, especially of pain, anxiety and depression may be insufficient. Regarding pain, many patients stated the need for adaptation of medication but also physiotherapy and general home based care. Other symptoms were often accepted, considered secondary to their underlying condition with no possibility for improvement. Physical symptoms had an important impact on the mood of patients, causing depression, increasing their dependency, leading to isolation and contributing to exhaustion of care givers. Anxiety was common among patients, which is also described in the literature as secondary to the uncertainties of living with their condition and approaching death [19]. In this regard, while only a few mentioned the need for specialised psychological support, perhaps due to cultural sensitivity, many found relief in faith and in the possibility to exchange their feelings, particularly with peers. Therefore, comprehensive symptom and pain management is essential to quality end of life care and support [20].

Thirdly, exhaustion of caregivers (essentially spouses or close relatives) emerged as significant, which has also been found elsewhere [21]. In addition, financial difficulties were recurrent and may put additional strain on caregivers [22]. While support to caregivers has been recognised as critical [23], their needs remain unmet. Patients and caregivers suggested home visits, both by volunteers, faith representatives and medical teams would reduce the burden and provide emotional support. Improving availability and affordability of consumables (e.g. diapers, urinary catheters) and equipment (e.g. wheelchairs) were also regularly mentioned in this regard. Our study highlighted how interlinked these factors are, stressing the importance of a comprehensive response that goes beyond medical palliative care.

Finally, information seems to be lacking about patient’s condition, prognosis and availability of services. Lack of understanding of their condition may result in feelings of helplessness with impeded decision making and control over their lives. Many patients did not ask for additional medical care, considering that symptoms could not be adequately managed. Indeed, provision of adequate information is crucial, as stated by Fallowfield [24]: “although truth hurts, deceit may well hurt more”. In addition, information about disease management is also needed in order for caregivers to support appropriately their sick family member [25].

### Strengths and limitations

Although the importance of end of life care and support is well established in many countries, palliative care services remain scarce in BiH and care is essentially provided by general practitioners [26]*.* Little information exists on existing services [27]. Here we have focused on the patients’ needs and perceptions to provide insights into the most adequate interventions [28]. The results of this study, in addition to further investigations about perceptions of care providers, social workers, volunteers and municipal authorities, will serve as the basis for developing acceptable, feasible and sustainable solutions, involving all stakeholders. Our participatory approach will help us to build a common understanding and a shared intervention strategy for quality patient centred end-of-life care and services in the municipality of Doboj. Based on the identification of needs and perceptions of both patients and providers to jointly design an intervention aimed at improving palliative care and support, our approach could be replicated successfully elsewhere to develop context specific strategies [29] and could help to reduce the significant burden carried by individuals, families and local communities [30]. Mobilisation of all actors and integration into the public health system is key to delivering sustainable services in settings with limited resources [31]. In addition, as stated by Gwyther [32], sharing of lessons learned from this initiative with policy makers at both the national and international level could trigger policy changes and promote integration of EoLC into BiH’s health programmes, which is a priority for the European Association for Palliative Care Initiative in Eastern Europe [33].

We acknowledge as limitation the possibility of selection bias of participants, as the FM teams of Doboj municipality had to list all patients which would qualify for end of life care. There might be patients recently discharged from hospital or not registered with FM teams who have not been listed and therefore, not selected. Nevertheless, the probability that patients are discharged without information to FM teams is quite small and more than 90% of the population is registered with FM. Patients unable to provide informed consent to participate, or with any disability that impedes communication were also excluded. The small sample size of our study may limit generalisation and external validity of the findings. However, we used a 2-tier, purposive sampling strategy and chose individuals to reflect the geographical and demographic characteristics of terminally ill patients. Another limitation might be that an appointed PHCC worker interviewed the patients. Patients may have been reluctant to make negative statements about care provided at the primary care level. On the other hand, the presence of a PHCC professional facilitated identification of patients needing immediate attention and referral to appropriate services.

## Conclusion

Our study, conducted in a resource limited setting where such issues are barely investigated, emphasised a wide gap between patients’ expectations and currently available care and services. While there is a need to improve symptoms management through training of health professionals including capacity building of nurses [34], better medication and provision of adequate material and devices, this will only tackle the patients’ most urgent concerns. Addressing the needs of patients at the end of their life, aiming at maintaining them with the best possible quality of life while providing appropriate support to their relatives, thus remains a much broader challenging task.

In order to ensure a comprehensive response to expressed needs, a coordinated inter-professional, inter-institutional and multi-stakeholder approach is required, including appropriate information to the patients and their caregivers about the disease, its stages and its management as well as adequate psychosocial, spiritual, administrative and financial support. The concept of nurse-led navigation / stewardship [35], that would provide patients with comprehensive services could be explored. A culture of anticipation should be adopted while providing end of life care and support.

## Additional file

Additional file 1: Interview guide. Questionnaire used to assess patient’s perceptions, needs and expectations. (DOCX 131 kb)

## Abbreviations

BiH: Bosnia-Herzegovina
DTHM: Division of Tropical and Humanitarian Medicine
EoLC: End of life care
FM: Family medicine
HUG: Hôpitaux Universitaires de Genève, Geneva University Hospitals
PHCC: Primary Health Care Centre
WHO: World Health Organisation
