# Supplementary material for: Addressing the needs of terminally-ill patients in Bosnia-Herzegovina: patients’ perceptions and expectations
Source: BMC Palliat Care. 2018 Nov 19;17:123. doi: 10.1186/s12904-018-0377-2 (PMC6245800; doi:10.1186/s12904-018-0377-2)
Supplement: Supplementary file 1 — Interview guide. Questionnaire used to assess patient’s perceptions, needs and expectations. (DOCX 131 kb) [file 12904_2018_377_MOESM1_ESM.docx]

Exploring End-of-Life Care and Support in Doboj Municipality

Questionnaire

Assessment of patients’ perceptions, needs and expectations

**Reminder - Objectives of the project:**

- ***To collect patients’ perceptions on existing support and services***
- ***To identify patients’ unmet needs and wants***
- ***To inspire ways to improve quality of life for patients suffering an incurable condition***

**Version of August 17th, 2016**

**(**SAP, ThV, ED, SD, RL with integration of comments from the field KGZ and palliative care team in Geneva and testing with the patient)

TABLE OF CONTENT

[INTRODUCTION – CONTEXT, RATIONALE AND PROPOSED APPROACH 3](#_Toc459279398)

[COVER PAGE TO BE DETACHED AND STORED BY PHCC DOBOJ 5](#_Toc459279405)

[COVER PAGE TO BE KEPT WITH FILLED QUESTIONAIRE AND STORED BY FONDACIJA FAMI 6](#_Toc459279406)

[QUESTIONNAIRE 7](#_Toc459279407)

[Question 1 – Are you informed about the course of your disease? 8](#_Toc459279408)

[Question 2 – In your opinion, according to your perception, are you treated with dignity? 9](#_Toc459279409)

[Question 3 – Do you experience problems related to your ability to be independent? 10](#_Toc459279410)

[Question 4 – How is your mood currently - Are you affected in your ability to enjoy life? 11](#_Toc459279411)

[Question 5 – Do you feel you have a low level of energy and feel tired or drowsy? 12](#_Toc459279412)

[Question 6 – Do you experience difficulties to breathe? 13](#_Toc459279413)

[Question 7 – Do you suffer from pain related to your disease? 14](#_Toc459279414)

[Question 8 – Do you have a lack of appetite? 15](#_Toc459279415)

[Question 9 – Are you nauseous recently? 16](#_Toc459279416)

[Question 10 – Do you experience digestive problems? 17](#_Toc459279417)

[Question 11 – Do you have particular fears or worries? 18](#_Toc459279418)

[Question 12 – Do you feel the need to sort out spiritual questions? 19](#_Toc459279419)

[Question 13 – Did your disease cause any financial difficulty to you or your relatives? 20](#_Toc459279420)

[Question 14 – Do you have difficulties dealing with administrative matters? 21](#_Toc459279421)

[Question 15 – Are you worried for the future of your family? 22](#_Toc459279422)

## INTRODUCTION – CONTEXT, RATIONALE AND PROPOSED APPROACH

Bosnia and Herzegovina (BiH) is a former Yugoslav republic situated at the heart of the Balkans.

With regard to healthcare in BiH, the former Yugoslavian health system was centrally planned and specialist-oriented with little or no emphasis placed upon patient centred care. Primary care physicians were mainly performing triage and providing little long term follow-up of patients (Perone et al., 2007). Most physicians were specialists, and most patients were eventually referred to a specialist. Nurses’ main activities consisted of collecting administrative data and giving injections.

Evidence suggests that a Primary Health Care (PHC) system yields better health outcomes than specialist and market-driven health systems (Starfield et al., 2005). Moreover, strong primary care based systems are cheaper to operate than those relying on multiple specialists. In “*Primary Care in the driver’s seat*” Boerma concludes that primary care-based systems are more cost effective (Boerma and Dubois, 2006:15).

In the framework of post conflict reconstruction and health care reform, strengthening primary health care based on the Family Medicine (FM) model has been a priority and is strongly supported by the ministries of health of both entities as well as by the international partners.

In 1999, the Council of Ministries of Bosnia-and-Herzegovina (BiH) established Family Medicine as a priority, reorganizing the health care system and reallocating resources to strengthen primary care. According to this plan, FM doctors should work in a patient-centred healthcare team, together with nurses being empowered and developing counselling activities and follow-up of patients with chronic and incurable diseases. The doctor and the nurse are to work in a team, sharing the same location, agenda and population of patients.

### Current situation in Doboj Municipality

The municipality of Doboj covers a population of approx. 80’000 persons. The municipal Primary Health Care Centre (PHCC) currently employs 31 FM teams covering the geographic territory of the whole municipality.

End of life care is provided on an ad-hoc basis and without an existing system or community network and support. End of life care activities are not documented and covered by the Health Insurance Fund (HIF). FM teams lack training in the field of end of life care, equipment for home visits, while transport remains an issue. Cooperation with other sectors and actors in community depend on individuals and their good will. Pain management is not standardised and access to medication such as opiates needs to be improved.

Leading causes of death in Doboj region are cardiovascular diseases (47%), cancer (22%), followed by metabolic and respiratory conditions (10%).

### Rationale

Primary Health Care (PHC) and Family Medicine reforms are well underway in BiH. A large part of BiH health services are moving away from specialist-centred, hospital-centred and doctor-centred healthcare towards comprehensive patient-cantered, community-based, integrated, continuous and multi-professional and multi-sectorial primary care. These changes have important implications, over and above for the patients. Early 2016, the management of the municipal Primary health care centre (PHCC) approached the *fami* Foundation in order to conduct a feasibility study with the intent to develop a comprehensive, affordable and sustainable range of end of life services and support in Doboj.

### Objective of the survey

In this study we will investigate the perceptions of patients on their needs and expectations in end of life care. We aim at a patient-centred approach, targeting quality of life, and offering a comprehensive, affordable and sustainable range of services and support.

We want to explore the different dimensions, needs and services associated with end-of-life care. Our thoughts are articulated around the quality of life of persons with an incurable condition.

In order to explore patients’ needs and expectations, we will start with individual patient interviews.

The topics/problems addressed in the interview are:

1. Information about the health condition, right / desire to know
2. Dignity, respect
3. Autonomy, physical, functional
4. Depression, anxiety
5. Tiredness/Drowsiness
6. Respiratory distress
7. Pain
8. Nutrition, appetite
9. Nausea/Vomiting
10. Digestive problems: Constipation/Diarrhoea
11. Distress, fear
12. Spiritual needs and believes
13. Financial issues
14. Administrative issues
15. Family/Future

Acceptability and feasibility of the collected proposals will be probed in a second stage by multidisciplinary focus groups (these will include representatives of patients, their families, health professionals, social workers and stakeholders involved in addressing end of life requirements).

Our participatory approach will help us achieve a common understanding, a shared intervention strategy and hopefully mobilize all resources and goodwill needed to develop comprehensive, affordable and sustainable quality patient centred end-of-life care in the municipality of Doboj.

We trust our exploratory study in the municipality of Doboj will serve as the basis for similar developments in other municipalities of BiH, the Balkan region and possibly beyond.

### Methods

This survey addresses 15 items in the form of a questionnaire, semi structured, combining open and closed questions and severity scales.
For each question patients will be asked to specify the following:

- Did he/she receive appropriate services to overcome his/her problem? (**Appreciation of current situation**)
- Should there be changes to the services made available in his/her situation? (**Proposed changes**)
- Should there be additional services made available in his/her situation? (**Proposed extensions/additions**)

The questionnaire will administered by one single interviewer, in the presence of a health care worker appointed by the PHCC manager (nurse or doctor).

In total 31 family medicine teams have registered approximately 900 patients with incurable diseases at an end of life stage in Doboj municipality.
Two patients per team, in total 62 patients, will be selected and individually interviewed for a maximum of 90 minutes per questionnaire. Patients will be of different age, gender, socio-economic status, education level and distance to PHCC (Dom Zdravlja / Ambulanta) and selected purposely to have a representative sample.

In case of advanced stages of confusion or significant disturbances of consciousness, identified during the interview, the interview will be stopped and appropriate measures taken.

### Ethics clearance

The proposal shall be submitted to the Geneva “*Commission Cantonale d’Ethique de la Recherche*” and the “*Ethical Board of the Primary Health Care Centre of Doboj*”.

### Data analysis

Completed questionnaires will be coded and introduced in EPI-DATA templates by Fondacija fami. Free texts provided in the respective spaces will be analysed separately and qualitatively. Analyses will be performed using SPSS.

## COVER PAGE TO BE DETACHED AND STORED BY PHCC DOBOJ

**Patient No**: __________

Date of interview: _____________

Place of interview: _____________

************************************

**Name and Last name of the patient**: ____________________

Gender: Male [ ] / Female [ ]

Date of birth: ____________________

Level of education: Primary school [ ], Secondary school [ ], Higher education [ ]

Distance from current living place to health care centre (PHCC / Field clinic):
(km) ___________ (minutes) ___________

Distance to the referral centre

(km) ___________ (minutes) ___________

************************************

**Name of interviewers**:

Name, Last name 1 __________________

Name, Last name 2 __________________

************************************

## COVER PAGE TO BE KEPT WITH FILLED QUESTIONAIRE AND STORED BY FONDACIJA FAMI

**Patient No**: __________

Date of interview: __________

Place of interview: __________

************************************

Patient gender: Male [ ] / Female [ ]

Patient date of birth: __________

Patient level of education: Primary school [ ], Secondary school [ ], Higher education [ ]

Distance from current living place of patient to health care centre (PHCC / Field clinic):
(km) __________ (minutes) __________

Distance to the referral centre:

(km) __________ (minutes) __________

************************************

**Name of interviewers:**

Name, Last name 1 __________________

Name, Last name 2 __________________

************************************

## QUESTIONNAIRE

### Question 1 – Are you informed about the course of your disease?

*(Patient is informed about his/her disease, access (to care and support depending on the needs? On financial means?), referrals (to whom, specify: PHC, oncologist, palliative care unit, hospital, physiotherapist, other specialist (specify), medications)*

| **Not at all** | **A little bit** | **Quite a bit** | **Completely** |
| --- | --- | --- | --- |
| **(--)** | **(-)** | **(+)** | **(++)** |

1. **What do you know about the course of your disease?**

____________________________________________________________________________________________________________________________________________________________________

1. **If your general condition worsens, would you want to be hospitalized or taken care at home?**

Hospitalized [ ] At home [ ]

1. **If you have been thinking where you would want to spend last days of your life, where would that be?**

____________________________________________________________________________________________________________________________________________________________________

1. **According to you, with your understanding of the course of your disease, do you have the feeling to be taken care of appropriately? please describe your answer**

__________________________________________________________________________________________________________________________________________________________________________________________________________________________________________________________________________________________________________________________________________________________________________________________________________________________________________________________________________________________________________

1. **According to you, with your experience, what kind of changes should there be to the care and the services made available to patients in your situation?**

__________________________________________________________________________________________________________________________________________________________________________________________________________________________________________________________________________________________________________________________________________________________________________________________________________________________

1. **What additional services should be made available to improve information of patients in your situation?**

________________________________________________________________________________________________________________________________________________________________________________________________________________________________________________________________________________________________________________________________________

### Question 2 – In your opinion, according to your perception, are you treated with dignity?

(Being honoured, respected, heard, listened and understood)

| **Never** | **Sometimes** | **Most of the time** | **Always** |
| --- | --- | --- | --- |
| **(--)** | **(-)** | **(+)** | **(++)** |

1. **Can you elaborate on your opinion?**

____________________________________________________________________________________________________________________________________________________________________________________________________________________________________________________________________________________________________________________________________________________________________________________________________________________________________________________________________________________________________________

1. **Should there be changes in the way dignity of the patient is considered in Doboj municipality and please elaborate your answer?**

__________________________________________________________________________________________________________________________________________________________________________________________________________________________________________________________________________________________________________________________________________________________________________________________________________________________________________________________________________________________________________________________________________________________________________________________________________________________________________________________________________________________________________________________________________________________________

1. **What additional services should there be made available to better respect dignity of the patients in your situation?**

________________________________________________________________________________________________________________________________________________________________________________________________________________________________________________________________________________________________________________________________________________________________________________________________________________________________________________________________________________________________________________________________________________________________________________________________________________________________________________________________________________

### Question 3 – Do you experience problems related to your ability to be independent?

(Does the patient have functional and physical autonomy; is he/she able to manage daily life activities?)

| **Not at all** | **A little bit** | **Quite a bit** | **Completely** |
| --- | --- | --- | --- |
| **(++)** | **(+)** | **(-)** | **(--)** |

1. **Did you receive appropriate support to overcome a lack of independence? Can you please elaborate your answer**

______________________________________________________________________________________________________________________________________________________________________________________________________________________________________________________________________________________________________________________________________________________________________________________________________________________________________________________________________________________________________________________________________________________________________________________________

1. **What changes to the services should there be made available to help patients in your situation?**

______________________________________________________________________________________________________________________________________________________________________________________________________________________________________________________________________________________________________________________________________________________________________________________________________________________________________________________________________________________________________________________________________________________________________________________________

1. **Could your level of autonomy / independence be improved?**

**Yes [ ] No [ ]**

**d) If Yes, how?**

__________________________________________________________________________________________________________________________________________________________________________________________________________________________________________________________________________________________________________________________________________________________________________________________________________________________________________________________________________________________________________________________________________________________________________________________________________________________________________________________________________________________________________________________________________________________________

### Question 4 – How is your mood currently - Are you affected in your ability to enjoy life?

(Check for depression or anxiety, check for causes (HADS scale). A depressive state is a temporary psychological state, which implies a decrease of tone and mood. If a depression settles over time, it may be a depression requiring specific attention. Depression is characterised by irritability, anxiety, insomnia, lack of momentum, energy and self-evaluation. Anxiety is defined by a feeling of imminent danger of unknown origin that combines emotional, cognitive, and behavioural symptoms. It can correspond to diffuse fear, constant stress when facing a situation or environment. It often manifests as a choking sensation like a lump in the throat or butterflies in the stomach)

| **Rarely** | **A few times** | **Very often** | **Most of the time** |
| --- | --- | --- | --- |
| **(++)** | **(+)** | **(-)** | **(--)** |

1. **Did you receive appropriate support? Can you please elaborate your answer**

______________________________________________________________________________________________________________________________________________________________________________________________________________________________________________________________________________________________________________________________________________________________________________________________________________________________________________________________________________________________________________________________________________________________________________________________

1. **Should there be changes to the services made available to help patients in your situation?**

______________________________________________________________________________________________________________________________________________________________________________________________________________________________________________________________________________________________________________________________________________________________________________________________________________________________________________________________________________________________________________________________________________________________________________________________

**c) Could your ability to enjoy life be improved?**

**Yes [ ] No [ ]**

**d) If Yes, how?**

__________________________________________________________________________________________________________________________________________________________________________________________________________________________________________________________________________________________________________________________________________________________________________________________________________________________________________________________________________________________________________________________________________________________________________________________________________________________________________________________________________________________________________________________________________________________________

### Question 5 – Do you feel you have a low level of energy and feel tired or drowsy?

(Tiredness is common in many diseases and appears frequently with a prevalence of 80%. It can manifest itself as physical exhaustion with difficulties to perform activities of the daily life, lack of energy or lack of desire. The causes can be pain, side effects of medication, infection, anaemia, cachexia, diabetes, dehydration, depression) Check for fatigue domains: emotional, cognitive, physical (weakness))

| **Rarely** | **A few times** | **Very often** | **Most of the time** |
| --- | --- | --- | --- |
| **(++)** | **(+)** | **(-)** | **(--)** |

1. **Did you receive appropriate care to overcome these symptoms? Can you please elaborate your answer**

______________________________________________________________________________________________________________________________________________________________________________________________________________________________________________________________________________________________________________________________________________________________________________________________________________________________________________________________________________________________________________________________________________________________________________________________

1. **Should there be changes to the care provided to patients in your situation?**

______________________________________________________________________________________________________________________________________________________________________________________________________________________________________________________________________________________________________________________________________________________________________________________________________________________________________________________________________________________________________________________________________________________________________________________________

**c) Should there be additional services made available to patients in your situation?**

__________________________________________________________________________________________________________________________________________________________________________________________________________________________________________________________________________________________________________________________________________________________________________________________________________________________________________________________________________________________________________________________________________________________________________________________________________________________________________________________________________________________________________________________________________________________________

### Question 6 – Do you experience difficulties to breathe?

(Dyspnoea and uncomfortable breathing sensation has an impact on anxiety and wellbeing of patients. Its prevalence is 30-75%. It can be related to tumour, infection, anaemia, asthenia, anxiety, secondary to treatment chemotherapy, heart failure, advanced COPD, restrictive diseases. Check for respiratory symptoms)

| **Rarely** | **A few times** | **Very often** | **Most of the time** |
| --- | --- | --- | --- |
| **(++)** | **(+)** | **(-)** | **(--)** |

1. **Did you receive appropriate care to overcome these symptoms? Can you please elaborate your answer**

______________________________________________________________________________________________________________________________________________________________________________________________________________________________________________________________________________________________________________________________________________________________________________________________________________________________________________________________________________________________________________________________________________________________________________________________

1. **Should there be changes to the care provided to patients in your situation?**

______________________________________________________________________________________________________________________________________________________________________________________________________________________________________________________________________________________________________________________________________________________________________________________________________________________________________________________________________________________________________________________________________________________________________________________________

1. **Should there be additional services made available to patients in your situation?**

______________________________________________________________________________________________________________________________________________________________________________________________________________________________________________________________________________________________________________________________________________________________________________________________________________________________________________________________________________________________________________________________________________________________________________________________

### Question 7 – Do you suffer from pain related to your disease?

(Assess pain and its impact, check for patients' barriers/perceptions to opioid use)

| **No pain** | **Moderate pain** | **Severe pain** | **Worst pain** |
| --- | --- | --- | --- |
| **(++)** | **(+)** | **(-)** | **(--)** |

1. **Did you receive appropriate care to overcome pain? Can you please elaborate your answer**

______________________________________________________________________________________________________________________________________________________________________________________________________________________________________________________________________________________________________________________________________________________________________________________________________________________________________________________________________________________________________________________________________________________________________________________________

1. **Should there be changes to the care provided to patients in your situation?**

______________________________________________________________________________________________________________________________________________________________________________________________________________________________________________________________________________________________________________________________________________________________________________________________________________________________________________________________________________________________________________________________________________________________________________________________

1. **Should there be additional services made available to patients in your situation?**

______________________________________________________________________________________________________________________________________________________________________________________________________________________________________________________________________________________________________________________________________________________________________________________________________________________________________________________________________________________________________________________________________________________________________________________________

### Question 8 – Do you have a lack of appetite?

(Check for loss of appetite, weight loss, eating related distress (MNA scale)

| **Most of the time** | **A lot of the time** | **From time to time, occasionally** | **Not at all** |
| --- | --- | --- | --- |
| **(--)** | **(-)** | **(+)** | **(++)** |

1. **Did you receive appropriate care / support to overcome lack of appetite? Can you please elaborate your answer**

______________________________________________________________________________________________________________________________________________________________________________________________________________________________________________________________________________________________________________________________________________________________________________________________________________________________________________________________________________________________________________________________________________________________________________________________

1. **Should there be changes to the care / support provided to patients in your situation?**

______________________________________________________________________________________________________________________________________________________________________________________________________________________________________________________________________________________________________________________________________________________________________________________________________________________________________________________________________________________________________________________________________________________________________________________________

1. **Should there be additional services made available to patients in your situation?**

______________________________________________________________________________________________________________________________________________________________________________________________________________________________________________________________________________________________________________________________________________________________________________________________________________________________________________________________________________________________________________________________________________________________________________________________

### Question 9 – Are you nauseous recently?

(Check for causes of nausea and vomiting, how patient deals with it. Nausea corresponds to disgust, without expelling contents of stomach through oesophagus and mouth. Nausea can be constant over the day, whereas vomiting is intermittent)

| **Rarely** | **A few times** | **Very often** | **Most of the time** |
| --- | --- | --- | --- |
| **(++)** | **(+)** | **(-)** | **(--)** |

**9.1) How severely do you feel these symptoms?**

| **Very mildly** | **Mildly** | **Severely** | **Very severely** |
| --- | --- | --- | --- |
| **(++)** | **(+)** | **(-)** | **(--)** |

1. **Did you receive appropriate care / support to overcome problem with nausea? Can you please elaborate your answer**

______________________________________________________________________________________________________________________________________________________________________________________________________________________________________________________________________________________________________________________________________________________________________________________________________________________________________________________________________________________________________________________________________________________________________________________________

1. **Should there be changes to the care / support provided to patients in your situation?**

______________________________________________________________________________________________________________________________________________________________________________________________________________________________________________________________________________________________________________________________________________________________________________________________________________________________________________________________________________________________________________________________________________________________________________________________

1. **Should there be additional services made available to patients in your situation?**

______________________________________________________________________________________________________________________________________________________________________________________________________________________________________________________________________________________________________________________________________________________________________________________________________________________________________________________________________________________________________________________________________________________________________________________________

### Question 10 – Do you experience digestive problems?

(Check for constipation (bowel movements 3 times of fewer per week) and or diarrhoea (3-4 loose stools a day or more): causes and severity))

| **Rarely** | **A few times** | **Very often** | **Most of the time** |
| --- | --- | --- | --- |
| **(++)** | **(+)** | **(-)** | **(--)** |

**10.1) What is the digestive problem?**

**Diarrhoea [ ] Constipation [ ] Both [ ]**

**10.2) How severely do you feel these symptoms?**

| **Very mildly** | **Mildly** | **Severely** | **Very severely** |
| --- | --- | --- | --- |
| **(++)** | **(+)** | **(-)** | **(--)** |

1. **Did you receive appropriate care / support to overcome digestive problems? Can you please elaborate your answer**

______________________________________________________________________________________________________________________________________________________________________________________________________________________________________________________________________________________________________________________________________________________________________________________________________________________________________________________________________________________________________________________________________________________________________________________________

1. **Should there be changes to the care / support provided to patients in your situation?**

______________________________________________________________________________________________________________________________________________________________________________________________________________________________________________________________________________________________________________________________________________________________________________________________________________________________________________________________________________________________________________________________________________________________________________________________

1. **Should there be additional services made available to patients in your situation?**

______________________________________________________________________________________________________________________________________________________________________________________________________________________________________________________________________________________________________________________________________________________________________________________________________________________________________________________________________________________________________________________________________________________________________________________________

### Question 11 – Do you have particular fears or worries?

(Check for causes of distress: fears, fear of death „unfinished business“, psychological/emotional distress)

| **Never** | **Occasionally** | **Quite often** | **Very often** |
| --- | --- | --- | --- |
| **(++)** | **(+)** | **(-)** | **(--)** |

1. **Did you receive appropriate support to overcome your fears or worries? Can you please elaborate your answer**

______________________________________________________________________________________________________________________________________________________________________________________________________________________________________________________________________________________________________________________________________________________________________________________________________________________________________________________________________________________________________________________________________________________________________________________________

1. **Should there be changes to the services made available to help patients in your situation?**

______________________________________________________________________________________________________________________________________________________________________________________________________________________________________________________________________________________________________________________________________________________________________________________________________________________________________________________________________________________________________________________________________________________________________________________________

1. **Should there be additional services made available to patients in your situation?**

__________________________________________________________________________________________________________________________________________________________________________________________________________________________________________________________________________________________________________________________________________________________________________________________________________________________________________________________________________________________________________________________________________________________________________________________________________________________________________________________________________________________________________________________________________________________________

### Question 12 – Do you feel the need to sort out spiritual questions?

(Check for spiritual and religious needs of patients, use of spiritual tradition and community for overall wellness)

| **Not at all** | **Occasionally** | **Often** | **Continuously** |
| --- | --- | --- | --- |

1. **Did you receive appropriate support to address these questions? Can you please elaborate your answer**

______________________________________________________________________________________________________________________________________________________________________________________________________________________________________________________________________________________________________________________________________________________________________________________________________________________________________________________________________________________________________________________________________________________________________________________________

1. **Should there be changes to the services made available to help patients in your situation?**

______________________________________________________________________________________________________________________________________________________________________________________________________________________________________________________________________________________________________________________________________________________________________________________________________________________________________________________________________________________________________________________________________________________________________________________________

1. **Should there be additional services made available to patients in your situation?**

__________________________________________________________________________________________________________________________________________________________________________________________________________________________________________________________________________________________________________________________________________________________________________________________________________________________________________________________________________________________________________________________________________________________________________________________________________________________________________________________________________________________________________________________________________________________________

### Question 13 – Did your disease cause any financial difficulty to you or your relatives?

(Check for financial loss due to sickness, cost of treatment, and cost for care givers, work lost. Does he/she has support from social welfare?)

| **Not at all** | **A little bit** | **Yes, but not too badly** | **Very definitely and quite badly** |
| --- | --- | --- | --- |
| **(++)** | **(+)** | **(-)** | **(--)** |

1. **Did you receive appropriate support and services to address financial issues? Can you please elaborate your answer**

______________________________________________________________________________________________________________________________________________________________________________________________________________________________________________________________________________________________________________________________________________________________________________________________________________________________________________________________________________________________________________________________________________________________________________________________

1. **Should there be changes to the support and/or services made available to help patients in your situation?**

______________________________________________________________________________________________________________________________________________________________________________________________________________________________________________________________________________________________________________________________________________________________________________________________________________________________________________________________________________________________________________________________________________________________________________________________

1. **Should there be additional support and/or services made available to patients in your situation?**

__________________________________________________________________________________________________________________________________________________________________________________________________________________________________________________________________________________________________________________________________________________________________________________________________________________________________________________________________________________________________________________________________________________________________________________________________________________________________________________________________________________________________________________________________________________________________

### Question 14 – Do you have difficulties dealing with administrative matters?

(Check for administrative barriers and causes)

| **Not at all** | **A little, but it doesn’t worry me** | **Yes, but not too badly** | **Very definitely and quite badly** |
| --- | --- | --- | --- |
| **(++)** | **(+)** | **(-)** | **(--)** |

1. **Did you receive appropriate support and services to address administrative issues? Can you please elaborate your answer**

______________________________________________________________________________________________________________________________________________________________________________________________________________________________________________________________________________________________________________________________________________________________________________________________________________________________________________________________________________________________________________________________________________________________________________________________

1. **Should there be changes to the support and/or services made available to help patients in your situation?**

______________________________________________________________________________________________________________________________________________________________________________________________________________________________________________________________________________________________________________________________________________________________________________________________________________________________________________________________________________________________________________________________________________________________________________________________

1. **Should there be additional support and/or services made available to patients in your situation?**

__________________________________________________________________________________________________________________________________________________________________________________________________________________________________________________________________________________________________________________________________________________________________________________________________________________________________________________________________________________________________________________________________________________________________________________________________________________________________________________________________________________________________________________________________________________________________

### Question 15 – Are you worried for the future of your family?

(Check for support for family members, where does he/she lives, with whom, who are caregivers, patient's concerns about his/her family)

| **Not at all** | **Not very much** | **Quite a lot** | **Very much indeed** |
| --- | --- | --- | --- |
| **(++)** | **(+)** | **(-)** | **(--)** |

1. **Did you receive appropriate support and services to address related questions? Can you please elaborate your answer**

______________________________________________________________________________________________________________________________________________________________________________________________________________________________________________________________________________________________________________________________________________________________________________________________________________________________________________________________________________________________________________________________________________________________________________________________

1. **Should there be changes to the support and/or services made available to help patients in your situation?**

______________________________________________________________________________________________________________________________________________________________________________________________________________________________________________________________________________________________________________________________________________________________________________________________________________________________________________________________________________________________________________________________________________________________________________________________

1. **Should there be additional support and/or services made available to patients in your situation?**

__________________________________________________________________________________________________________________________________________________________________________________________________________________________________________________________________________________________________________________________________________________________________________________________________________________________________________________________________________________________________________________________________________________________________________________________________________________________________________________________________________________________________________________________________________________________________

### Question 16 – We would like to sincerely thank you for the participation in the survey. May we quote your answers?

**Yes [ ] No [ ]**

If yes, are there any responses you don't want us to quote?

________________________________________________________________________________________________________________________________________________________________________________________________________________________________________________________________________________________________________________________________________________________________________________________________________________________________________________________________________________________________________________________________________________________________________________________________________________________________________________________________________________
